# Key Factors Governing Microbial Community in Extremely Acidic Mine Drainage (pH <3)

**DOI:** 10.3389/fmicb.2021.761579

**Published:** 2021-11-30

**Authors:** Ye Huang, Xiu-Tong Li, Zhen Jiang, Zong-Lin Liang, Pei Wang, Zheng-Hua Liu, Liang-Zhi Li, Hua-Qun Yin, Yan Jia, Zhong-Sheng Huang, Shuang-Jiang Liu, Cheng-Ying Jiang

**Affiliations:** ^1^State Key Laboratory of Microbial Resources, Institute of Microbiology, Chinese Academy of Sciences, Beijing, China; ^2^School of Life Science, University of Chinese Academy of Sciences, Beijing, China; ^3^School of Minerals Processing and Bioengineering, Central South University, Changsha, China; ^4^Key Laboratory of Biometallurgy of Ministry of Education, Central South University, Changsha, China; ^5^National Engineering Laboratory for Hydrometallurgical Cleaner Production Technology, Institute of Process Engineering, Chinese Academy of Sciences, Beijing, China; ^6^Zijin Mining Group Company Limited, Fujian, China; ^7^School of Metallurgy and Environment, Central South University, Changsha, China

**Keywords:** acid mine drainage, mineralogy, microbial diversity, co-occurrence, biogeochemical function potential

## Abstract

The microbial community of acid mine drainage (AMD) fascinates researchers by their adaption and roles in shaping the environment. Molecular surveys have recently helped to enhance the understanding of the distribution, adaption strategy, and ecological function of microbial communities in extreme AMD environments. However, the interactions between the environment and microbial community of extremely acidic AMD (pH <3) from different mining areas kept unanswered questions. Here, we measured physicochemical parameters and profiled the microbial community of AMD collected from four mining areas with different mineral types to provide a better understanding of biogeochemical processes within the extremely acidic water environment. The prominent physicochemical differences across the four mining areas were in SO_4_^2−^, metal ions, and temperature, and distinct microbial diversity and community assemblages were also discovered in these areas. Mg^2+^ and SO_4_^2−^ were the predominant factors determining the microbial structure and prevalence of dominant taxa in AMD. *Leptospirillum*, *Ferroplasma*, and *Acidithiobacillus* were abundant but showed different occurrence patterns in AMD from different mining areas. More diverse communities and functional redundancy were identified in AMD of polymetallic mining areas compared with AMD of copper mining areas. Functional prediction revealed iron, sulfur, nitrogen, and carbon metabolisms driven by microorganisms were significantly correlated with Mg^2+^ and SO_4_^2−^, Ca^2+^, temperature, and Fe^2+^, which distinguish microbial communities of copper mine AMD from that of polymetallic mine AMD. In summary, microbial diversity, composition, and metabolic potential were mainly shaped by Mg^2+^ and SO_4_^2−^ concentrations of AMD, suggesting that the substrate concentrations may contribute to the distinct microbiological profiles of AMD from different mining areas. These findings highlight the microbial community structure in extremely acidic AMD forming by types of minerals and the interactions of physicochemical parameters and microbiology, providing more clues of the microbial ecological function and adaptation mechanisms in the extremely acidic environment.

## Introduction

Acid mine drainage (AMD), or acid rock drainage, is a natural or man-made extremely acidic environment formed by spontaneous oxidation of pyrite and other sulfide minerals in contact with oxygen and water (Simate and Ndlovu, 2014). The generation of AMD is usually accelerated with the participation of microorganisms (Pronk and Johnson, 1992; Johnson et al., 1993). AMD is strongly acidic, rich in sulfur, and has a high concentration of metals, which can contaminate the peripheral and groundwater environment and cause serious pollution problems (Akcil and Koldas, 2006; Carlier et al., 2020; Pakostova et al., 2020).

Microorganisms and their metabolism in AMD have attracted much attention in recent years (Johnson, 2012; Gao et al., 2020; Liang et al., 2020). The relatively simple community structure of AMD means it serves as a model system for studying the evolution and adaption of microorganisms and their abilities to shape the environment (Li et al., 2014). AMD can also provide clues for the study of the origin of life and ancient microbial communities similar to its ecology (Amils et al., 2011; Havig et al., 2017). Culture-dependent and culture-independent microbial community analysis methods have been widely used in the study of AMD, generating data on AMD microbial diversity, community function, and the interactions between microbes and the environment (Kuang et al., 2013; Hua et al., 2015; Huang et al., 2016).

The microbial diversity of AMD is far less than that of other environments, yet some microbes thrive in the harsh AMD environment (Gupta et al., 2019; Johnson and Quatrini, 2020). Culture-dependent analysis indicated that the isolated iron- and sulfur-oxidizing bacteria *Acidithiobacillus* and the iron-oxidizing bacteria *Leptospirillum* are instrumental in the dissolution of sulfide minerals and the formation of AMD. The rapid development of high-throughput next-generation sequencing technologies and advanced bioinformatics tools have revealed rare taxa and unknown groups which were previously overlooked, including Micrachaeota and Parvarchaeota (Chen et al., 2018), which are members of Archaeal Richmond Mine Acidophilic Nanoorganisms (ARMAN; Krause et al., 2017). These studies enhanced knowledge of the diversity of microorganisms present in extremely acidic environments and uncovered the environmental distribution, adaption, and function of such microorganisms in the generation of AMD. The effects of physicochemical factors on the diversity and composition of the microbial community in these extremely acidic environments have been discussed and how extremophilic microorganisms respond to environmental variations have been further explored. The factor contributing most to structuring AMD microbial communities was reported to be pH despite the long-distance separation and variation of substrate types (Kuang et al., 2013; Liu et al., 2014). However, the pH values of AMD in these studies were in a wide range (from pH 0.5 to 7.3). Consequently, further exploration of the influences of environmental variables on microbial diversity of extremely acidic AMD (0<pH<3) is required. As we know, there is a complex relationship between the physicochemical factors and the microbiology of the mineral environment. Some indigenous bacteria and archaea could oxidize the specific ores to generate SO_4_^2−^ and release metal ions; pH was reduced at the same time. Therefore, mineral type should also be considered as a factor that shapes the AMD microbial community. Some studies suggested that environmental heterogeneity in the AMD environment might have a significant influence on the microbial communities present (Amaral Zettler et al., 2011; Liu et al., 2014). Microbial community structure was driven by the chemical composition of minerals, indicating the selective pressure of the chemical elements on microbial populations (Gleeson et al., 2006). Specific microbial communities were recruited on the surface of minerals according to mineral physicochemical properties and acted as metabolically active members in the process of formation or dissolution of minerals (Kelly et al., 2016; Valdespino Castillo et al., 2018). Furthermore, carbon content varies on the surface of different minerals (Fortin et al., 2000) and thus might have a decisive effect on the survival of microorganisms. In this study, AMD samples from four mining areas in southern China and Myanmar with different mineral types were collected and characterized on physicochemical factors microbial diversity and community structure in the samples. The detailed site descriptions are as following: (1) Zijinshan (ZJS) copper mine (116.38 N, 25.19 E) was one of the largest refractory low-grade Cu ore, mainly contained chalcocite, chalcopyrite, enargite, and covellite, with pyrite as associated minerals (Liu et al., 2016). (2) Monywa (MYW) copper mine (95.1 N, 22.1 E) has been operated for about 23years with a process of multi-lift heap leaching; the main mineral of MYW is chalcocite (average Cu grade of 0.33%; Jia et al., 2016). (3) Bainiuchang polymetallic mine (MZ; 103.46 N, 23.28 E) is an important part of the Sn polymetallic ore belt in South China. The orebodies of the area are clastic-carbonate rocks which contain abundant mineral elements of Zn, Pb, Cu, Ag, and Sb, combining with sulfur (Liu et al., 2007). (4) Dabaoshan (DBS) polymetallic mine (113.72 N, 24.52 E) is a large-scale and open-pit polymetallic mineral deposit in South China (Zhang et al., 2019). The upper of the main ore body is mainly composed of limonite, while the lower body appears to be metal sulfide ores and associated bismuth, tungsten, molybdenum, gold, and silver metal ores (Wan et al., 2009). The main geochemical factors affecting the community structures were elucidated, and how the microbial populations were influenced by these factors was examined. Co-occurrence relationships between archaeal and bacterial, core and rare, and autotrophic and heterotrophic groups were explored for the different mining areas, and niche divisions dominated by contributing factors were further investigated. Prediction of biogeochemical cycles driven by microorganisms was performed, aiming to obtain a more comprehensive understanding of the ecological function of microbial assemblages and their essential roles in the formation of AMD.

## Materials and Methods

### Study Site, Sample Collection, and Physicochemical Analyses

A total of 24 AMD water samples with different mineralogy were collected from four mining areas; the detailed information of sampling locations is provided in Supplementary Table 1. Nine water samples named ZJA11, ZJA12, ZJA13, ZJA21, ZJA22, ZJA23, ZJA31, ZJA32, and ZJA33 were collected from AMD collection pond A1, A2, and A3 of the heap bioleaching area of ZJS copper mine in Shanghang, Fujian Province, in October 2018. The three acid mine water wells had a continuously high-temperature inflow (about 40°C). Three extremely acidic water samples (approximate pH 0.7), MYW1, MYW2, and MYW3, were collected from the MYW copper mine in Myanmar. Six AMD samples, named BD1, BD2, and BD3, and CD1, CD2, and CD3, were collected from two naturally formed acid mine water pits in the Bainiuchang polymetallic mining area (MZ) in Mengzi City, Yunnan Province, in September 2017. Six water samples, C1W, C3W, C4W, C5W, C6W, and C9W, were collected from water pits C1, C3, C4, C5, C6, and C9 in the DBS polymetallic mine in Guangdong Province in October 2018. The temperature, pH, and conductivity of each sampling point were measured by a portable water quality multi-parameter meter (Star A329, Thermo Orion, United States). Iron (Fe^2+^, Fe^3+^) and SO_4_^2−^ concentration were determined using a portable colorimeter (DR/890, HACH, United States) based on the o-phenanthroline spectrophotometry (Environmental Protection Industry Standard of China, HJ/T 345-2007) and turbidimetric method, respectively. Approximately 10ml water samples were digested by solution composed of nitric acid and hydrochloric acid (8:4v/v) using a microwave digestion instrument (TOPwave, Analytik Jena, Germany) and heated on an electric hot plate to remove the acid. After diluting to an appropriate concentration, the solution was loaded onto an inductively coupled plasma-optical emission spectrometry (ICP-OES) analyzer (Optima 5300 DV, PerkinElmer, United States) to determine element contents. Hierarchical clustering was used to determine the similarity/dissimilarity of the physicochemical parameters of the samples, and Principal Component Analysis (PCA) was performed to retain the most important environmental factors using R 3.6.3 software package vegan (Dixon, 2003), FactoMineR (Lê et al., 2008), and factoextra.

### DNA Extraction and 16S rRNA Gene Amplicon Sequencing

For DNA extraction, water samples were filtered through a sterile microporous filter membrane (PES, Φ47–50mm, 0.22μm, PALL, United States) using a solvent filter and a vacuum pump. Genomic DNA was extracted from the filters by following the manual of the PowerMax Soil DNA Isolation Kit (Qiagen, United States). The DNA was dissolved in tris hydrochloric acid buffer (pH 8.0) and frozen at −20°C. The V4–V5 region of the 16S rRNA gene of almost all Bacteria and Archaea in the samples was amplified using the primer set 515F (5'-GTGYCAGCMGCCGCGGTAA-3') and 926R (5'-CCGYCAATTYMTTTRAGTTT-3'). PCR reactions were performed using KAPA HiFi HotStart ReadyMix (KAPA, Woburn, MA, United States) with amplification conditions comprising a pre-denaturation step at 95°C for 3min, then 25 cycles of denaturation at 98°C for 20s, annealing at 55°C for 15s, and extension at 72°C for 15s, followed by a final extension at 72°C for 1min. The resulting PCR products were purified by gel electrophoresis, the DNA concentration was determined by Qubit fluorometer (Qubit4, ThermoFisher Scientific, United States), and the sequencing adapter was connected to complete the library construction. Sequencing of 16S rRNA gene amplicons was carried out on an Illumina MiSeq PE250 platform by Beijing Boaohuijiu Biotechnology Co., Ltd.

### Processing of Pyrosequencing Data and Statistical Analysis

Clean sequencing reads of each sample were obtained by dividing datasets into subsets according to the sample tags. Single-ended sequences were merged using USEARCH v11, and primers were trimmed using Cutadapt v2.3 (Edgar, 2010; Martin, 2011). After removing low-quality sequences and chimeric and filtering singletons, high-quality reads were denoised to obtain zero-radius operational taxonomic units (zOTUs) using USEARCH v11. In Qiime 2, the primer set 515F/926R was used to train the RDP species classifier (Bolyen et al., 2019). Taxonomic classification was performed against the Silva 138 16S rRNA gene database (December 2019 release). Data transformation and manipulation were performed using R packages phyloseq v1.28.0, microbiome v1.6.0^1^ and microeco v0.3.4 (McMurdie and Holmes, 2013; Liu et al., 2021a). According to the annotation results, mitochondrial and chloroplast sequences were removed. To decipher the metabolic and ecologically relevant functions of the AMD microbial communities, taxon abundance profiles were converted and mapped into putative functional abundance profiles using FAPROTAX (Louca et al., 2016).

Community compositions at every taxonomic level were counted, and alpha diversity indexes of each sample were calculated. zOTUs with a relative abundance ≥1% were defined as abundant zOTUs and those with a relative abundance <0.01% were defined as rare zOTUs (Xue et al., 2020). Similarities and differences in communities were determined by principal coordinate analysis (PCoA) of Bray–Curtis distances obtained using vegan v2.5.6 (Dixon, 2003). The Mantel test was used to quantify the relationship between environmental factors and microbial assembly. Distance-based redundancy analysis (db-RDA) was conducted on core zOTUs and selected environmental parameters (SO_4_^2−^, temperature, Fe^2+^, Mg^2+^, and Ca^2+^) to identify possible correlations between environmental gradients and community changes. Values of *p* for multiple comparisons were adjusted using the false discovery rate of the Benjamini–Hochberg method. All statistical analyses were performed using the R v3.6.3 software package.

### Co-occurrence Network Construction

Microbial co-occurrence networks were constructed to characterize the microbial niches with different mineralogy. The correlation was calculated using the OTU that appeared in at least five samples and had more than 10 average sequences in the sample. SParse InversE Covariance estimation for Ecological Association and Statistical Inference (SPIEC-EASI) was performed and correlations with coefficients <0.3 were filtered (Kurtz et al., 2015). Positive correlations were exclusively focused on based on their mathematical interpretation and biological meaning. Global network properties were calculated to describe the topology using igraph packages in R (Csardi and Nepusz, 2006), and the networks were visualized using Gephi.

## Results

### Physicochemical Profiles of AMD

**Figure 1:**
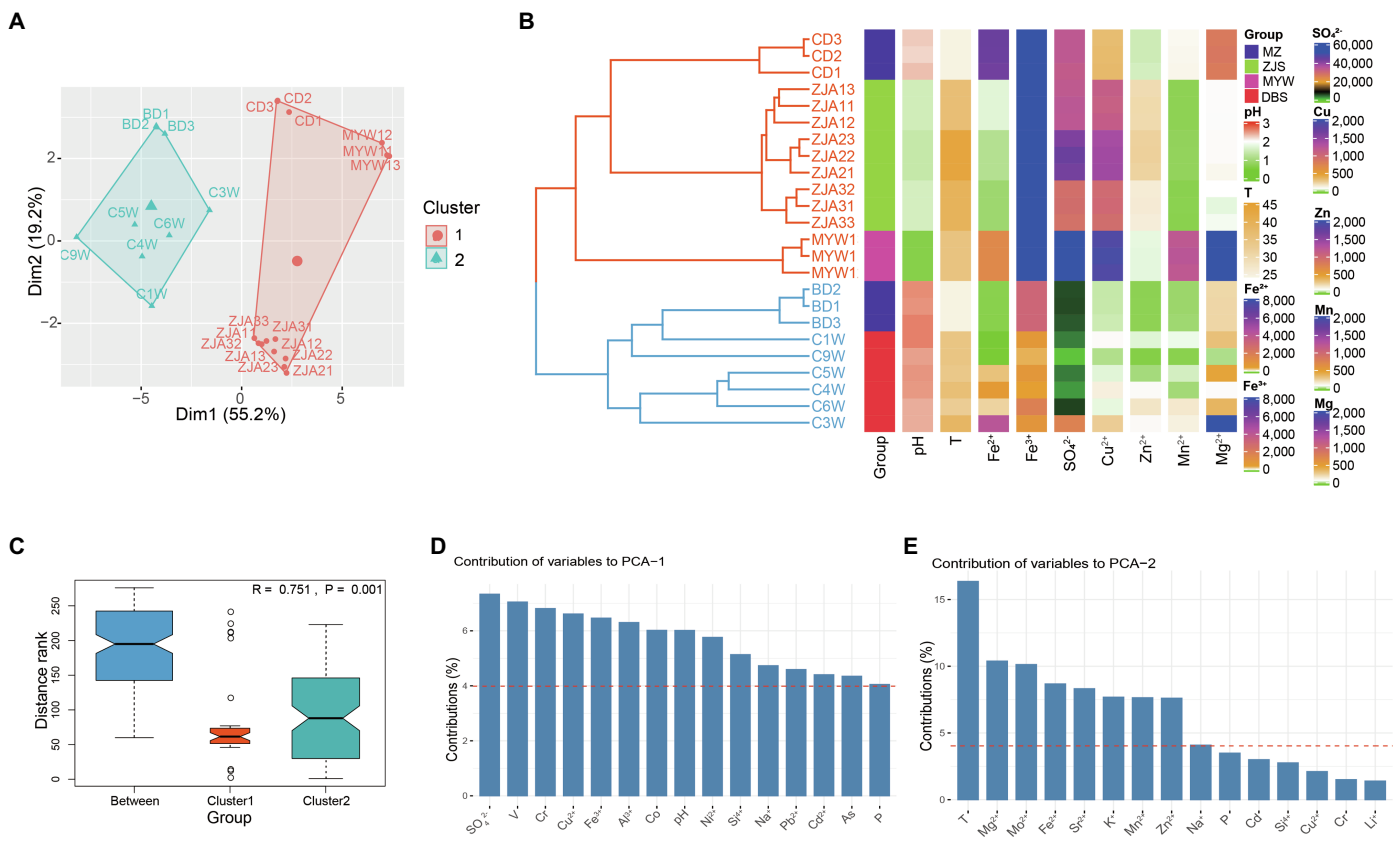
Differences in physicochemical properties of acid mine drainage (AMD) from different mining areas. **(A)** Principal components analysis of AMD samples. **(B)** Hierarchical clustering of samples based on all environmental factors (*k*=2, Euclidean distance and average clustering method was used) and heatmaps for important environmental factors. T represents temperature; units for ion concentrations are mgL^−1^. **(C)** Anosim analysis of differences between groups and within groups. Here, group means cluster generated by hierarchical clustering. Contribution of environmental variables to PCA1 **(D)** and PCA2 **(E)** of PCA analysis.

The AMD samples collected from DBS, MZ, ZJS, and MYW were characterized by extremely low pH (0.7–2.59), high ferrous and ferric ion concentrations (ranging from 1.575 to 6,482mgL^−1^ and from 409.18 to 36,504mgL^−1^, respectively), and high sulfate concentration (1,721–161,220mgL^−1^; Supplementary Table 1). Dissolved metals including Al^3+^, Mg^2+^, Cu^2+^, and Mn^2+^ were also abundant in the samples with concentrations ranging between 40.58–12,346, 44.64–3,576, 45.66–1,923.2, and 15.24–1,229.6 mgL^−1^, respectively. Other metal species present in substantial amounts included Zn, As, B, and Cr. Ba, Cd, Li, Mo, Ni, Pb, and Sr were measured as trace elements (<5 mgL^−1^) in most of the AMD samples except for the high concentration of Ni (~33.45 mgL^−1^) in MYW1-3. All chemical parameters were analyzed and included in PCA analysis and Hierarchical Clustering (Figures 1A,B). Hierarchical clustering analysis showed that AMD samples were divided into two main groups according to physicochemical parameters. One cluster included all samples from the ZJS and MYW sites, which were both copper mines. Another cluster comprised most polymetallic mines samples from the MZ and DBS sites. Heatmap for the environmental factors of AMD confirmed that the cluster of all samples from the ZJS and MYW copper mines did differentiate from the cluster of MZ and DBS polymetallic mines (Figure 1B). Cluster 1 had higher concentrations of Fe^3+^, SO_4_^2−^, Cu^2+^, and Zn^2+^ than cluster 2. PCA showed that the two clusters were significantly different from each other (Anosim, *R*=0.751, *p*=0.001; Figures 1A,C). PCA axes 1 explained 55.2% variance of samples, which was mainly contributed by SO_4_^2−^ and metals including V, Cr, Cu^2+^, and Fe^3+^ (Figure 1D). PCA axes 2 explained 19.2% variance, which was mainly contributed by temperature, Mg^2+^, Mo^2+^, and Fe^2+^ (Figure 1E). These results demonstrated that SO_4_^2−^, metal ions, and temperature were the factors that contributed most to the variation of samples from the different mining areas.

### Community Composition and Microbial Diversity in AMD

**Figure 2:**
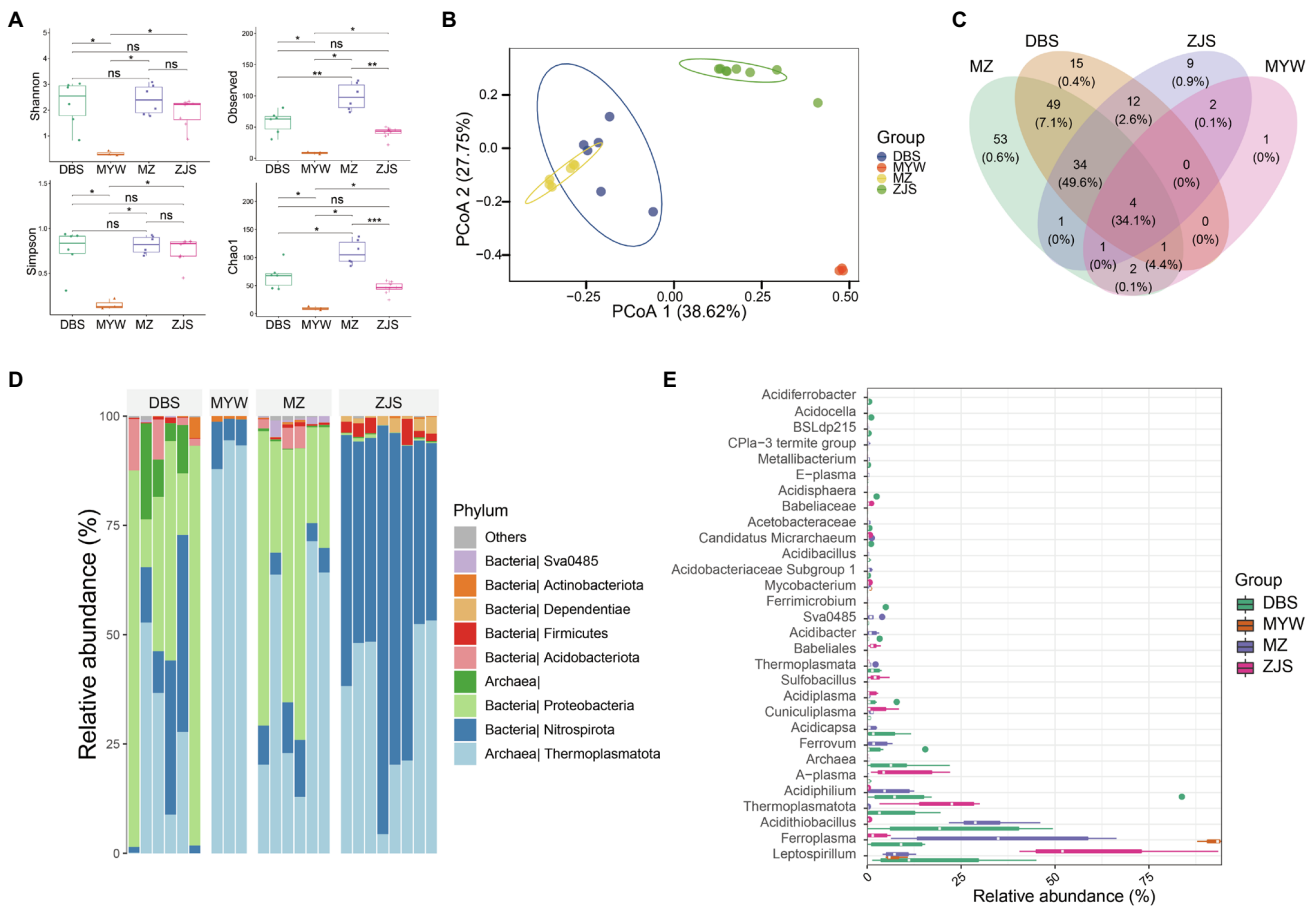
Dissimilarities of AMD microbial communities of four mining areas. **(A)** Alpha-diversity of AMD shown by the Shannon diversity index. **(B)** Principal coordinates analysis based on Bray–Curtis distance between samples. **(C)** Venn analysis of zero-radius operational taxonomic units (zOTUs) detected in four AMDs. **(D)** Relative abundance of microorganisms detected in AMD samples at the phylum level. **(E)** Top 50 taxa, at the genus level, in four AMD microbial communities. ^*^p < 0.05, ^**^p < 0.01, ^***^p < 0.001.

Sequencing and data manipulation generated 611,447 quality sequences, with an average of 26,184 sequences and a range of 5,894–49,612 sequences per sample (samples with <5,000 sequences were filtered). A total of 184 zOTUs were obtained after denoising and filtering. Sample clustering based on microbial community composition also demonstrated that the AMD samples clustered together according to the mining areas (Figure 2). Comparative analysis of the alpha diversity index including Shannon, Simpson, Chao1, and Observed OTUs (Richness) revealed that samples of MZ, DBS, and ZJS had significantly higher microbial diversity than samples of MYW (Figure 2A). All of the alpha diversity indexes (with an average Shannon index score of 0.31 and Observed OTUs number of 7) indicated that microbial diversity was extremely low in AMDs of MYW. Although the Shannon and Simpson indexes were similar for MZ, DBS, and ZJS AMDs, MZ AMD had the highest observed OTUs and Chao1 indices, followed by DBS AMD, which means that microbial community richness was higher in MZ and DBS AMDs. Compositional similarities and dissimilarities of microbial communities were further explored using PCoA of Bray–Curtis distances. The total interpretation of the first two axes, PCo1 and PCo2, was 65.37%. There were significant differences between the microbial communities across mining areas (Figure 2B). Prevalence analysis of zOTUs showed that four shared zOTU were detected in all AMD samples (accounting for 34.1% of the total sequences), while there were 54, 15, 9, and 1 unique zOTUs in samples of MZ, DBS, ZJS, and MYW, respectively (Figure 2C).

From the total sequences generated, over 97.8% of reads (the average among samples) could be classified under the Kingdoms of Bacteria and Archaea, but approximately 2.2% remained unclassified at the domain level. Among the classified sequences, 13 phyla were identified, with Thermoplasmatota, Nitrospirota, and Proteobacteria (average relative abundance of 41, 28.2, and 24.3%, respectively) representing the most abundant phyla (Figure 2D). At the genus level, more than 52.5% of the total sequences were distributed in the two most dominant genera in all AMD samples; these genera were *Leptospirillum* and *Ferroplasma* (Figure 2E). ZJS contained the highest abundance of *Leptospirillum*, while MYW was dominated by *Ferroplasma*. *Acidithiobacillus* were particularly richer in MZ and DBS (with an average relative abundance of 27.1%) than in ZJS and MYW (with an average relative abundance of 0.07%). The relative abundance of other phylotypes varied across the mining areas. *Acidiphilium*, *Ferrovum*, *Acidicapsa*, and *Acidibacter* were more abundant in MZ and DBS (average relative abundance of 12.5, 0.3, 0.8, and 0.9%, respectively) than in ZJS and MYW. ZJS samples had the highest relative abundances of *A-plasma* (9.3%), *Sulfobacillus* (2.3%), *Cuniculiplasma* (2.4%), and a genus affiliated with Babeliaceae (2.9%) which all occurred in lower proportions in other AMD (0.3, 0.17, 0.81, and 0%, respectively). *Ferrimicrobium*, *Acidisphaera*, *Metallibacterium*, *Acidocella*, and *Acidiferrobacter* were exclusively detected in DBS AMD.

### Environmental Factors Determining AMD Microbial Community Structure

**Figure 3:**
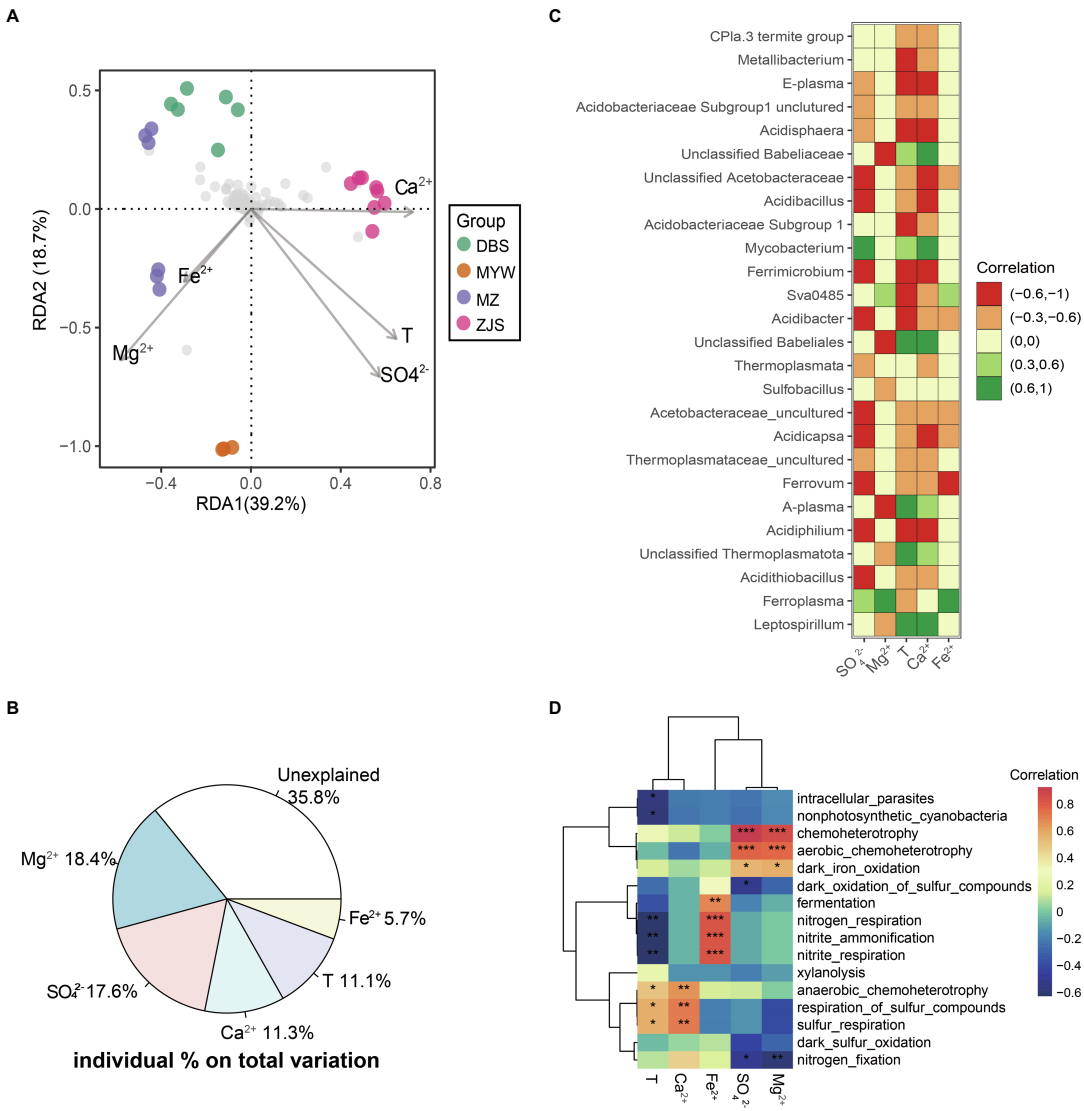
Environmental factors determining AMD microbial community structure. **(A)** db-RDA of microbial communities. Abundance data were Hellinger-standardized. **(B)** Variation partition of key contributing factors on dissimilarity of microbial communities. **(C)** Pearson correlations (*p*<0.01) between abundant taxa and most important environmental factors. **(D)** Heatmaps of Pearson correlations between predicted functions of microbial communities and determining environmental factors in AMD. ^*^p<0.05, ^**^p<0.01, ^***^p<0.001.

A significant correlation between physicochemical variation and microbial community dissimilarity in AMD was confirmed by the Mantel test (*r*=0.521, *p*=0.001). Furthermore, db-RDA was performed to identify possible correlations between physicochemical characteristics and microbial community patterns of the four mining areas (Figure 3A). Environmental factors with multicollinearity were filtered based on the value of Variance Inflation Factors (VIF). The step model based on the lowest Akaike Information Criterion (AIC) was performed to determine the fewest influencing factors but with the best interpretation. In this db-RDA analysis, variations of five optimal physicochemical parameters—SO_4_^2−^, Mg^2+^, Ca^2+^, temperature, and Fe^2+^—collectively explained 64.2% of the microbial community dissimilarity (Figure 3B). Among them, Mg^2+^ and SO_4_^2−^ were the environmental factors with the highest explanation values of 18.4% (*r*^2^=0.44, *p*=0.001) and 17.6% (*r*^2^=0.49, *p*=0.001), respectively, followed by Ca^2+^ with 11.3% (*r*^2^=0.35, *p*=0.005), temperature with 11.1% (*r*^2^=0.41, *p*=0.006), and Fe^2+^ with 5.7% (*r*^2^=0.11, *p*=0.307; Figure 3B).

At the level of individual dominant taxa, the genus *Leptospirillum* was positively correlated with temperature and Ca^2+^ but negatively correlated with Mg^2+^ (Figure 3C). Positive correlations of *Ferroplasma* with Fe^2+^, Mg^2+^, and SO_4_^2−^ were observed, while *Acidithiobacillus* showed negative correlations with temperature, Ca^2+^, and SO_4_^2−^ (Figure 3C). A genus affiliated with Thermoplasmatota, *A*-*plasma*, and a genus affiliated with Babeliales were also positively correlated with temperature and Ca^2+^ but negatively correlated with Mg^2+^ like the genus *Leptospirillum*. *Acidiphilium*, *Ferrimicrobium*, *Acidibacillus*, *Acidisphaera*, a genus affiliated with uncultured Acidobacteriaceae Subgroup1, *E*-*plasma*, *Ferrovum*, *Acidicapsa*, *Acidibacter*, and a genus affiliated with uncultured Acidobacteriaceae were all negatively correlated with temperature, Ca^2+^, and SO_4_^2−^. *Ferrovum*, *Acidicapsa*, and a genus affiliated with uncultured Acidobacteriaceae were negatively correlated with Fe^2+^, while Sva0485 was positively correlated with Fe^2+^. In summary, the microbial communities in AMD were mainly influenced by the environmental factors of Mg^2+^, SO_4_^2−^, Ca^2+^, temperature, and Fe^2+^.

### Co-occurrence of zOTUs in AMD

**Figure 4:**
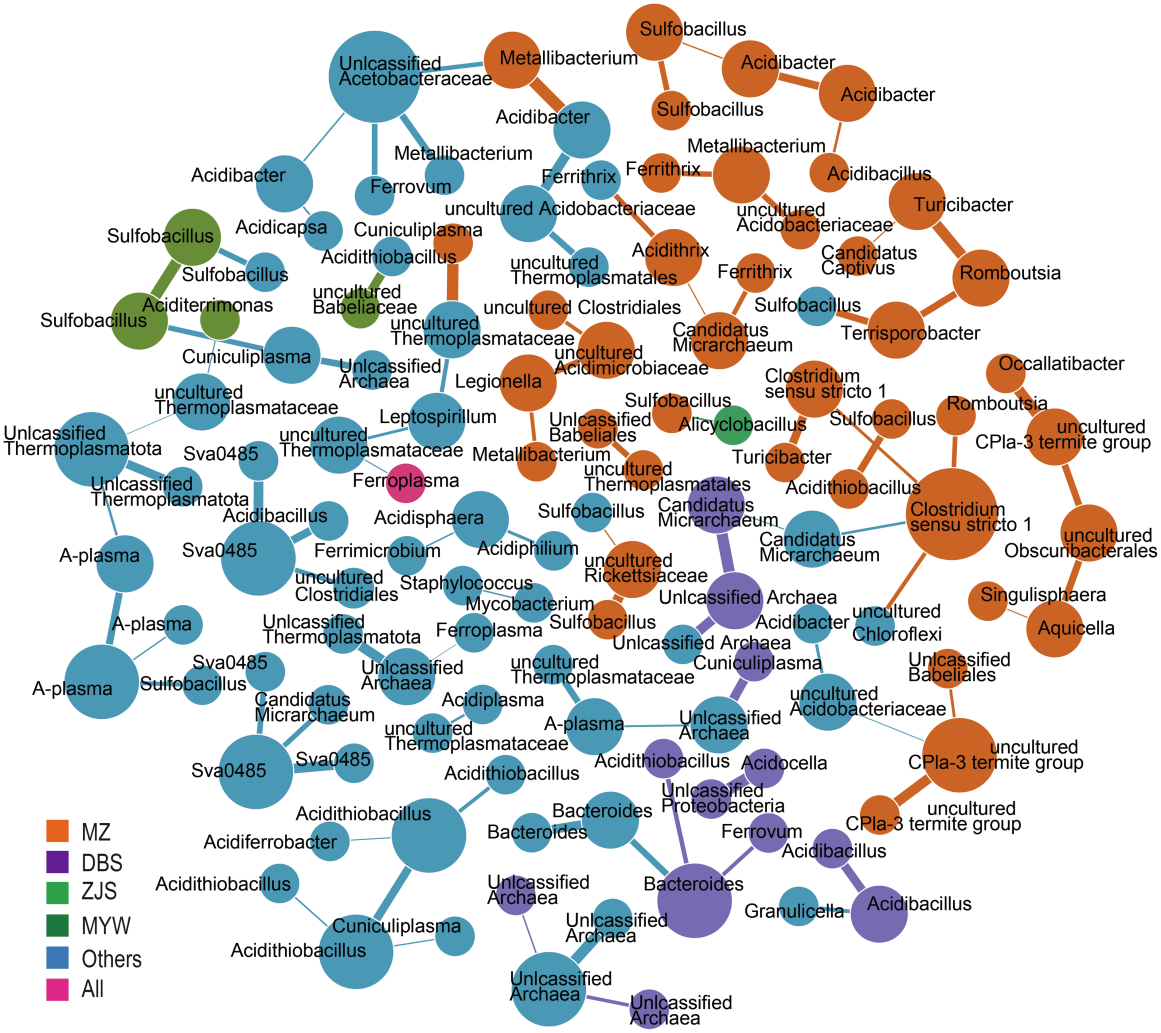
Co-occurrence network analysis. Colors represent prevalence patterns of nodes. mining area (MZ), Dabaoshan (DBS), Monywa (MYW), and Zijinshan (ZJS) mean nodes occurred exclusively in that mining area. All represented nodes are presented in all mining areas.

To decipher the co-occurrence pattern of the AMD microbial community, correlations between microorganisms at the zOTU level of samples from the four mining areas were calculated. Strong correlations (*r*>0.3) were retained to build co-occurrence networks (Figure 4). A total of 121 nodes and 92 edges were generated. Based on within-module connectivity (Zi) and among-module connectivity (Pi), nodes were classified as network hubs (Zi>2.5, Pi>0.6), module hubs (Zi>2.5, Pi<0.6), connectors (Zi<2.5, Pi>0.6), and peripherals (Zi<2.5, Pi<0.6; Fan et al., 2018). The results implied a sparse and loose network because 116 peripherals, five connectors, no network hub, and no module hub were identified. Co-occurred zOTUs varied from the mining sites. There were 40, 12, 4, and 1 node(s) exclusively detected in MZ, DBS, ZJS, and MYW, respectively, and unique correlations existed among these nodes. In MZ AMD, the sulfur-oxidizing bacterial genus *Sulfobacillus* was correlated with iron- and sulfur-oxidizing bacteria *Acidithiobacillus*, iron-reducing bacteria *Acidibacter*, and uncultured Rickettsiaceae. Rare zOTUs that were only detected in MZ were found to be related to each other. For example, uncultured CPla-3 termite group was closely related to unclassified Babeliales, uncultured Acidobacteriaceae Subgroup1, and uncultured *Obscuribacter*, while *Turribacter* was correlated with *Clostridium sensu stricto* 1, *Romboutsia*, and “*Candidatus* Captivus.” For zOTUs that occurred in at least two sites, correlations were observed between archaea and archaea (such as unclassified Thermoplasmatota and unclassified archaea), archaea and bacteria (such as uncultured Thermoplasmataceae and *Leptospirillum*, and *Cuniculiplasma* and *Acidithiobacillus*), sulfur- and/or iron-metabolizing and heterotrophic bacteria (such as sva0485 and *Acidibacillus*, and *Ferrovum* and unclassified Acetobacteraceae), and heterotrophic and heterotrophic bacteria (such as *Acidisphaera* and *Acidiphilium*).

### Metabolic Potential of Microbial Community and Individual Organisms in AMD

To investigate the potential biogeochemical cycles mediated by microorganisms of different mining areas, microbial community metabolic functions were predicted using FAPROTAX. Across the four mining areas, the metabolisms associated with anaerobic and aerobic chemoheterotrophy and dark iron oxidation were dominant in the AMD environment (Supplementary Table 2 and Figure 3D). Sulfur metabolisms including oxidation of sulfur compounds, sulfur and sulfur compound respiration, and dark sulfur oxidation were predicted in the MZ, DBS, and ZJS sites but not in the MYW area. *Acidithiobacillus*, *Leptospirillum*, *Ferroplasma*, *Acidiferrobacter*, *Ferrimicrobium*, and *Ferrithrix* were responsible for iron oxidation in MZ and DBS, while *Ferroplasma* was the only taxa predicted as an iron-oxidizing microorganism in MYW. In MZ, *Leptospirillum* and *Clostridium sensu stricto* 1 were involved in nitrogen metabolism, including nitrogen fixation, nitrite ammonification, and nitrite respiration. Nitrogen fixation was mainly driven by *Leptospirillum* in DBS and ZJS.

Correlations between potential metabolism functions and environmental factors were also calculated (Figure 3D). SO_4_^2−^ and Mg^2+^ were significantly positively correlated with aerobic chemoheterotrophy and dark iron oxidation but negatively correlated with dark oxidation of sulfur compounds and nitrogen fixation. Anaerobic chemoheterotrophy and sulfur respiration were positively correlated with temperature and Ca^2+^. Fermentation and nitrogen metabolisms (including nitrogen respiration, nitrite ammonification, and nitrite respiration) were significantly positively related to Fe^2+^ but negatively related to temperature.

## Discussion

### AMD of Different Mineral Types Showed Distinct Physicochemical Characteristics

Being mined and abandoned mines that contained varying amounts and types of metal sulfides minerals have the potential to generate acidic mine discharge water that contain high concentrations of sulfate and metals and metalloids. The physicochemical characteristics of AMD are subject to considerable variation (Johnson, 2003). In this study, the physicochemical characteristics of the AMD generated from four mining areas with different types of mines were distinct but were all extremely acidic and with high concentrations of SO_4_^2−^, iron, and other toxic metal ions. Similar to previous reports on other AMD systems (Lukhele et al., 2019), Al^3+^, Mg^2+^, Cu^2+^, Mn^2+^, Zn^2+^, As, B, and Cr were detected as abundant metal species, and Ba^2+^, Cd^2+^, Li^+^, Mo^2+^, Ni^2+^, Pb^2+^, and Sr^2+^ were detected as trace elements in all AMD samples of the current study. However, AMD from copper (ZJS and MYW) and polymetallic (MZ and DBS) mining areas presented different metal concentration profiles; AMD of the copper sulfide mines contained a higher concentration of metal ions (except for Li^+^ and Sr^2+^) compared with polymetallic mines. Lower pH and higher SO_4_^2−^ concentration were also observed in copper sulfide AMD. MYW and ZJS are both low-grade copper sulfide deposits; the dominant mineral of them was chalcocite, with acid-insoluble pyrite as associated minerals. AMD from them was generated during the bioheap-leaching process. In the case of acid-insoluble sulfide minerals oxidation, more ferric iron and protons are produced (Johnson, 2003). In contrast, MZ and DBS are polymetallic mines containing more complex ores, AMD from them were formed by multimineral dissolution. That might be the reason why AMD of MYW and ZJS contained higher concentrations of metal ions with lower pH compared to DBS and MZ. In practice, SO_4_^2−^ concentration is an important tracer to distinguish AMD-impacted waterbodies from unimpacted waterbodies (Havig et al., 2017). Statistical analysis in the current study also indicated that SO_4_^2−^ was the most important contributing factor determining the environmental divergence of AMD. Congruent with previous research (Liu et al., 2014), results from the present study demonstrated that acidity and concentrations of SO_4_^2−^ and metals of AMD were largely dependent on the composition of the exposed minerals; therefore, AMD from copper sulfide mines show more extreme characteristics than that of polymetallic mines.

### Mg^2+^, SO_4_^2−^, Ca^2+^, Temperature, and Fe^2+^ Affected the Structure and Potential of AMD Microbial Communities

Community dissimilarity was further explored along environmental gradients. Mg^2+^, SO_4_^2−^, Ca^2+^, temperature, and Fe^2+^ were the most contributing factors that affected the microbial community in AMD environments. In many previous studies, pH was the key determinant relating to and structuring AMD microbial communities (Kuang et al., 2013; Teng et al., 2017). pH can reflect the stages of the mineral oxidation process (Chen et al., 2013, 2014) and define the ecological niches of closely associated bacteria and archaea (Belnap et al., 2011). However, these previous studies captured a wide range of pH gradients from very low (below 2) to neutral (about 7) and thus may have underestimated and veiled the influences of other environmental factors on microbial communities (Aguinaga et al., 2018). The current study focused on AMD with a narrow range of pH at a low level (pH 0.7–2.59) and detailed the factors regulating extremely acidic AMD microbial populations. It has been known that pH has a strong influence on the dissolution of metals from ores, especially under low pH (<3.0). Accordingly, in our study, AMDs (such as MYW) contained higher concentrations of metal ions with lower pH, which might successively affect the microbial diversity. Nevertheless, microorganisms living in AMD appeared to have high resistance to highly toxic metals, which is why highly toxic metals such as copper, zinc, cadmium, and so on did not show a significant relationship with the microbial community. The research of Walton and Johnson (1992) indicated that the influence of pH change on the abundances of microorganisms was probably less significant than the change of substrate concentrations, such as Magnesium.

Mg^2+^ and Ca^2+^ were previously suggested to exert essential roles on aquatic microbial community composition in terrestrial aquifers (Fillinger et al., 2019) like hypersaline lakes (Podell et al., 2014), high-altitude Andean lakes (Núñez Salazar et al., 2020), and hot springs (Huang et al., 2013). Calcium ions participate in a variety of microbial functions, including heat shock, chemotaxis, and biodegradation. Furthermore, Ca^2+^ concentration in the aquatic environment is inextricably linked to the pH value and is related to inorganic carbon metabolism driven by microorganisms (Zavarzin, 2002). Indeed, almost all core taxa were affected by Ca^2+^ concentration in the current study. SO_4_^2−^ and Fe^2+^ were also identified as important factors that contributed to the variance of microbial communities in the present study. SO_4_^2−^ and Fe^2+^ were the main ions produced during the sulfide mineral oxidation process and likely reflected the degree of minerals dissolved (Teng et al., 2017). Most heterotrophic microorganisms were negatively related to SO_4_^2−^concentration, and this might be why the abundance of these groups decreased significantly during the later stage of mineral oxidation. In addition, SO_4_^2−^ was directly correlated with the metabolism of sulfur-oxidizing microbes (Dopson and Johnson, 2012) and Fe^2+^ concentration largely affects iron-oxidizing microorganisms (Walton and Johnson, 1992). The current study demonstrated that SO_4_^2−^ and Fe^2+^ may have correlations with the nitrogen and carbon cycles driven by microorganisms in the AMD environment. This may be a direct influence since these essential geochemical processes were mainly attributed to key iron- and sulfur-oxidizing microorganisms like *Leptospirillum* or/and *Acidithiobacillus*, which are strongly affected by SO_4_^2−^ and Fe^2+^. The hazardous metals such as arsenic were generally believed to have a significant influence on the microbial community structure. Nevertheless, our results showed only a few microorganisms that had strong negative correlations with dissolved arsenic (Supplementary Figure 1). Microorganisms living in AMD appeared to have high resistance with highly toxic arsenic (Laroche et al., 2018). The temperature was observed as another important contributing factor that influenced the community structure and prevalence of the dominant microbes. Temperature is an essential condition for the growth of microorganisms. Different species were reported to dominate the microbial community on ore surfaces at different temperatures during the bioleaching process (Wang et al., 2018). Furthermore, temperature-regulated microbial iron and arsenic oxidation in AMD (Tardy et al., 2018) was correlated with the microbial population and acid generation (Jia et al., 2018). Individual microbes likely have distinct functional responses to variations in temperature. Elevated temperature significantly upregulated the carbon fixation and amino-acid metabolism proteins of *Leptospirillum* in AMD biofilms collected from the Richmond Mine (Mosier et al., 2015). This was consistent with the findings of the current study that *Leptospirillum* was positively correlated with temperature. Archaea, mainly affiliated with phylum Thermoplasmatota, were previously found to be dominant in AMD with low-to-moderate temperatures and were considered significant contributors to carbon- and iron-cycling (Korzhenkov et al., 2019). In the present study, Thermoplasmatota-related taxa, including *Ferroplasma* and *E-plasma*, negatively correlated with temperature, demonstrating the potential association between carbon and iron metabolism with temperature, and that the niche of dominant taxa is divided by temperature.

### AMD Microbial Community Assembly Was Largely Influenced by Mineral Types

The role of mineral type on AMD microbial communities was explored in the current study. AMD of polymetallic mines had higher microbial diversity than copper mines, and many rare taxa were exclusively detected in polymetallic AMD, suggested a more diverse community. Heterotrophic iron-metabolizing bacteria *Acidiphilum*, reported to can reduce soluble- and solid-phase ferric iron, was significantly more abundant in polymetallic AMD (12% relative abundance) than in copper AMD (relative abundance of 0.00086%). Other heterotrophic microorganisms, including *Acidisphaera*, an unclassified genus within Acetobacteraceae, *Acidibacter*, *Acidibacillus*, and *Acidicapsa* were also more prevalent in the polymetallic AMD environment. Furthermore, functional redundancies were revealed in polymetallic AMD. Low-abundance taxa might serve as the functional pool whose members are recruited during the specific acidification process to generate AMD. For example, the acidophilic iron-oxidizing microorganisms *Ferrimicrobium*, *Ferrithrix*, and *Ferrovum*, as well as *Acidiferrobacter*, were exclusively observed in polymetallic AMD (Supplementary Table 3). In addition to *Leptospirillum*, *Clostridium* detected as rare taxa in polymetallic AMD were also identified as nitrogen-metabolizing bacteria. Together, these results demonstrated that the prevalence of rare taxa in AMD was mainly influenced by the AMD mineralogy type and highlighted the contributing roles of rare taxa groups to AMD (Liu et al., 2014).

### Dominant Taxa Prevalence Was Related to Mineral Type in AMD

*Leptospirillum*, *Ferroplasma*, and *Acidithiobacillus* were identified as the core genera in AMD as they are detected globally in AMD sites and play important roles in the dissolution of sulfide minerals and the generation of AMD (Huang et al., 2016; Xiao et al., 2016). In the current study, the dominance of *Leptospirillum* and *Ferroplasma* in AMD of all four mining sites was reported, but *Acidithiobacillus* was only abundant in polymetallic AMD. *Leptospirillum* and *Ferroplasma* have been frequently discovered in extremely acidic environmental conditions, like biofilm floating on acidic water or growing on the surface of an ore body (Bond et al., 2000a,b; Lo et al., 2007). *Leptospirillum* and *Ferroplasma* were always found to coexist in environments where the pH was below 1 (Tyson et al., 2004; Demergasso et al., 2005). *Leptospirillum* is extremely acidophilic, chemolithotrophic bacteria, with the capabilities of dark iron oxidation, growth by carbon fixation *via* the reductive tricarboxylic acid (rTCA) cycle (Levican et al., 2008), and nitrogen fixation (Goltsman et al., 2013). The paucity of the iron uptake system may explain the dominance of these bacterial taxa in extremely low pH environments with soluble iron concentrate more than 14gL^−1^ (Battaglia et al., 1994; Osorio et al., 2008). *Ferroplasma* was described as a chemomixotrophic, cell-wall-lacking, iron-oxidizing archaea and was predicted to assimilate carbon and nitrogen sources from outside the cell (Dopson et al., 2005). With a tetraether lipid membrane monolayer structure, *Ferroplasma* was profoundly acid-tolerant and heavy metal resistant (Golyshina and Timmis, 2005), especially it could endure more than 36gL^−1^ iron in AMD of MYW. *Acidithiobacillus* is psychrophilic to mesophilic (Nordstrom et al., 2015) and thus may dominate the AMD microbial communities at lower temperatures as indicated by the results of the current study. A negative correlation with SO_4_^2−^ suggested the essential role of *Acidithiobacillus* in the sulfur oxidation of AMD (Wang et al., 2019).

### Network Analysis Implied Unexpected Co-occurrence Patterns Among AMD Microbial Communities

Network analysis is an efficient tool to decipher the sophisticated interactions of microbial communities and provides constructive information for experimental verification of interactions among microbes. Bioinformatic tools have been developed to calculate the relationships within a microbial community according to the variations in microbial abundance profiles. Taxa count data of the AMD microbial communities in the current study were overdispersed and had excess zeros. To fit the count data with overdispersion and excess zeros and minimize false positives of the correlations, SPIEC-EASI was applied to infer the AMD microbial ecological networks. Sparse but robust interactions were present among microbes across mining areas. There were distinct mineral-type-related correlations and specific relationships among various heterotrophic bacteria in polymetallic AMD. Rare taxa linked to mammalian diseases, including *Legionella*, *Mycobacterium*, *Turicibacter*, *Terrisporobacter*, and *Romboustia*, were correlated with typical AMD groups such as *Metallibacterium* and *Sulfobacillus*. It remains unclear that why these neutrophilic heterotrophs could appear in an extremely acidic environment like AMD. Previous researches highlighted the importance of Horizontal Gene Transfer events (HGT) in the environmental adaptions of some eukaryotes like *Galdieria sulphuraria* and prokaryotes like *Acidiphilium* and *Alicyclobacillus* to the AMD environment (Schonknecht et al., 2013; Li et al., 2020; Liu et al., 2021b). Genes acquired horizontally are involved in ecologically important processes including heat tolerance, toxic metals resistance, and metabolites transportation and metabolism, etc. Our results suggested possible HGT and/or metabolic coupling among these taxa. HGT may be a common strategy for microorganisms to adapt to the harsh and acidic environment. Distinct interactions between sulfur- and/or iron-metabolizing and heterotrophic microorganisms were also found, which implied indispensable roles of heterotrophic microorganisms in the geochemical cycles for key elements in AMD.

## Conclusion

This study focused on the physicochemical characteristics and microbial community features of the extremely acidic AMD environments (pH below 3.0) and identified correlations between them. Harsh conditions of AMD with high acidity, high concentrations of sulfate and metal ions, and distinct microbial community structures were revealed. Our results showed that SO_4_^2−^ and metal ions (especially for Mg^2+^) were the key contributing factors that differentiated samples from the different mining areas and controlled the microbial diversity and communities structure in these areas, which also revealed geochemical element cycles driven by microorganisms were significantly correlated with Mg^2+^, SO_4_^2−^, Ca^2+^, temperature, and Fe^2+^ in AMD, regardless of the pH of these environments. Greater knowledge of the microbiological compositions and their potential function in these ecosystems, as well as the intricate interactions between the environment and microbial community assembly, will doubtless lead to the more efficient application of these extremely microorganisms in treating AMD.

## Data Availability Statement

The datasets generated for this study have been deposited in the National Omics Data Encyclopedia (NODE; https://www.biosino.org/node/) with accession code OEX013755 and can also be found in the NCBI under accession number PRJNA640232.

## Author Contributions

S-JL and C-YJ designed the study. YJ provided the MYW samples, and Z-SH provided the ZJS samples for the study. Z-LL and PW collected MZ, ZJS, and DBS AMD samples. X-TL and ZJ characterized the physicochemical properties of the samples. YH analyzed the data and wrote the manuscript. Z-HL, L-ZL, and H-QY provided technical support for data analysis. All authors contributed to the article and approved the submitted version.

## Funding

This work was supported by the National Nature Science Foundation of China (91851206, 41877345, and 31670124), CAS – NSTDA Joint Research Project (153211KYSB20200039), the Joint Funds of Innovation Academy for Green Manufacture, Chinese Academy of Sciences (IAGM2020C24), and the CAS Engineering Laboratory for Advanced Microbial Technology of Agriculture, Chinese Academy of Sciences (KFJ-PTXM-016).

## Conflict of Interest

Z-SH is employed by Zijin Mining Group company Limited.

The remaining authors declare that the research was conducted in the absence of any commercial or financial relationships that could be construed as a potential conflict of interest.

The handling editor declared a shared affiliation with several of the authors C-YJ, S-JL, YH, X-TL, ZJ, Z-LL, PW, and YJ at time of review.

## Publisher’s Note

All claims expressed in this article are solely those of the authors and do not necessarily represent those of their affiliated organizations, or those of the publisher, the editors and the reviewers. Any product that may be evaluated in this article, or claim that may be made by its manufacturer, is not guaranteed or endorsed by the publisher.

## Footnotes

^1^ https://github.com/microbiome/microbiome
