# Supplementary figures and images for: Key Factors Governing Microbial Community in Extremely Acidic Mine Drainage (pH <3)
Source: Front Microbiol. 2021 Nov 30;12:761579. doi: 10.3389/fmicb.2021.761579 (PMC8670003; doi:10.3389/fmicb.2021.761579)

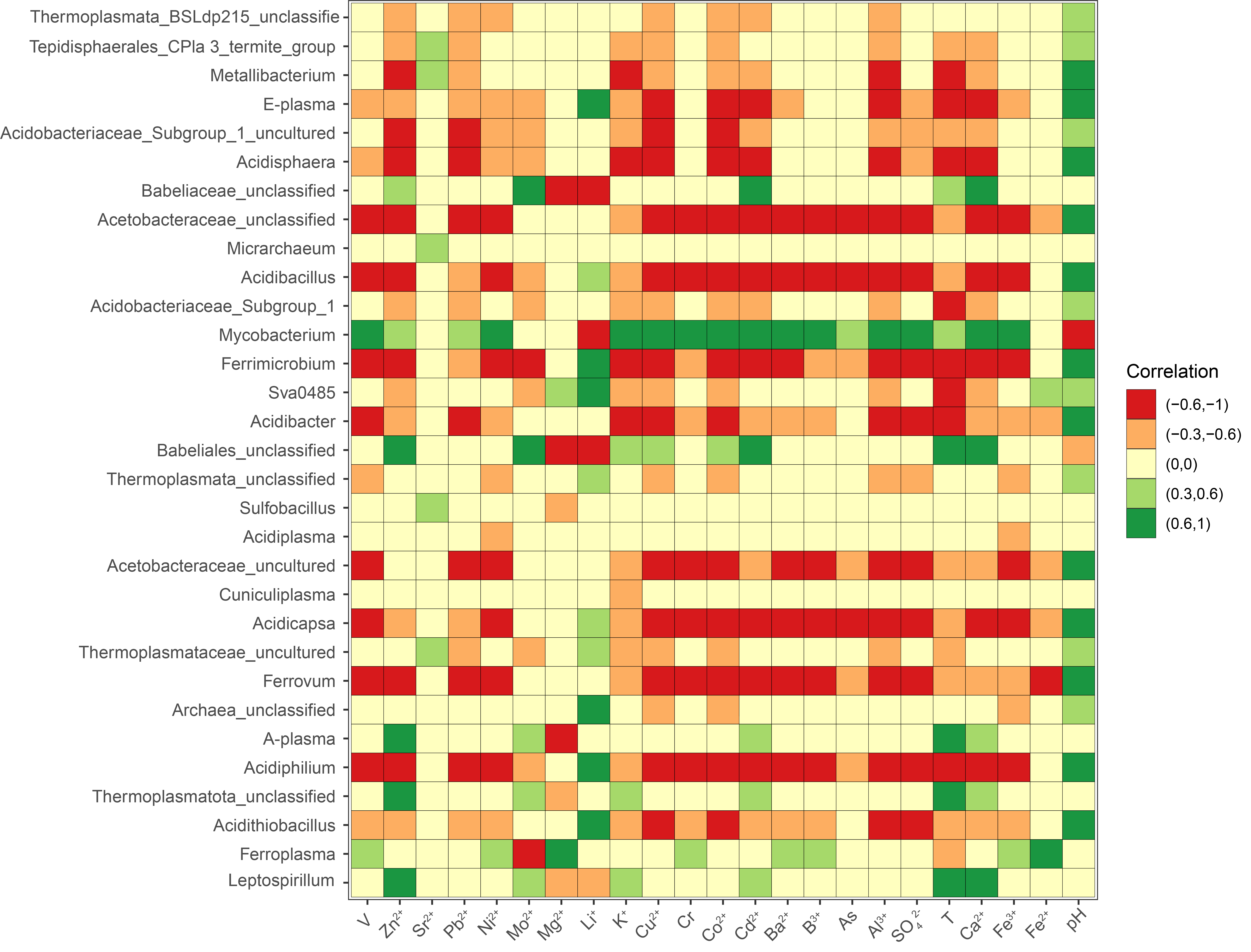

Supplement: Supplementary file 4 [file Image_1.tif]
